# Wearable Sleep-Stage Classification: A Systematic Analysis of Signal Modalities, Representations, and Multimodal Fusion

**DOI:** 10.3390/s26185746

**Published:** 2026-09-10

**Authors:** Unaza Tallal, Rupesh Agrawal, Shruti Kshirsagar

**Affiliations:** 1 School of Computing, Wichita State University, 1845 Fairmount St., Wichita, KS 67260, USA; 2 Health Informatics Department, Northern Kentucky University, Highland Heights, KY 41099, USA; agrawalr1@nku.edu

**Keywords:** sleep staging, photoplethysmograms, wearable sensors, accelerometry, short-time Fourier transform, handcrafted features, deep learning, multimodal fusion

## Abstract

Accurate sleep staging is essential for characterizing sleep architecture, yet conventional polysomnography limits scalable and long-term sleep monitoring. Wearable sensors offer a practical alternative, but their performance depends on the physiological modalities used and how the signals are represented. This study investigates the effects of signal modality, feature representation, and sleep-stage granularity on subject-independent wearable sleep staging. Using DREAMT, we compare handcrafted features, short-time Fourier transform (STFT) representations, and their fusion across three-, four-, and five-class tasks. Handcrafted features were modeled using a CNN–BiGRU framework, while STFT representations of BVP and accelerometry were learned using a deep time–frequency model. All DREAMT experiments were evaluated using five-fold subject-independent cross-validation, with Macro-F1 and Cohen’s κ emphasized to account for class imbalance. We further evaluated optical pulse signal-based classification using the larger MESA Sleep cohort. STFT improved class-balanced performance over handcrafted features, while BVP was the strongest individual STFT modality, particularly for REM. For the three-, four-, and five-class tasks, handcrafted BVP + ACC achieved Macro-F1 scores of 0.5057, 0.4005, and 0.3366, respectively, compared with 0.6404, 0.5083, and 0.4376 using STFT. Hybrid fusion achieved Macro-F1 scores of 0.6476, 0.5108, and 0.4464, respectively. More complex fusion strategies did not improve Macro-F1 over simple concatenation. In MESA, increasing the cohort from 100 participants to the full eligible cohort improved pulse-based sleep-stage classification, although N1 and N3 remained comparatively difficult. Overall, the results show that effective signal representation and complementary physiological information are more important than simply increasing the number of modalities or model complexity.

## 1. Introduction

Sleep staging provides important information about sleep architecture and is widely used to assess sleep quality and sleep-related disorders. Standard sleep staging divides a recording into Wake, rapid eye movement (REM), and non-REM stages N1, N2, and N3 according to established scoring criteria [1]. Polysomnography (PSG) remains the clinical reference standard because it combines electroencephalography, electrooculography, electromyography, and other physiological measurements. However, PSG requires specialized equipment and trained personnel, which limits its practicality for repeated and long-term sleep monitoring. Wearable devices provide a less intrusive alternative for continuous sleep monitoring. Modern wrist-worn devices can record blood volume pulse (BVP), accelerometry (ACC), electrodermal activity (EDA), and skin temperature, as well as cardiac measures such as heart rate (HR) and interbeat intervals (IBI) [2]. These modalities capture different aspects of sleep physiology. Cardiac signals reflect autonomic changes, while accelerometry primarily captures movement. EDA and skin temperature provide additional information related to sympathetic and thermoregulatory activity [3]. Recent wearable studies increasingly combine PPG and accelerometry for multi-stage sleep classification, although performance varies considerably across sensor configurations and evaluation settings [4]. A key challenge is how to represent wearable signals. Traditional approaches use handcrafted features to characterize pulse morphology, cardiac variability, movement, electrodermal activity, and temperature. Deep learning approaches can instead learn representations from raw signals or time–frequency transformations. Short-time Fourier transform (STFT) representations preserve changes in spectral content over time and provide structured inputs for deep models. However, it remains unclear whether STFT representations consistently provide an advantage over handcrafted physiological features and whether combining the two representations captures complementary information.

The contribution of each modality may also depend on sleep-stage granularity. A signal that effectively separates Wake, NREM, and REM may provide less information when NREM is divided into N1, N2, and N3. Similarly, adding more modalities or using more complex fusion strategies may introduce redundant information without improving class-balanced performance. Performance may further depend on cohort size and signal-acquisition conditions. These factors make it difficult to determine whether improvements arise from the physiological modality, signal representation, fusion strategy, or available training data. In this study, we systematically investigate how physiological modality, signal representation, and sleep-stage granularity affect subject-independent wearable sleep staging. We use DREAMT for multimodal experiments with wrist-recorded BVP, ACC, EDA, skin temperature, HR, and IBI [2]. We compare handcrafted and STFT-based representations, quantify the contribution of individual and combined modalities, and evaluate whether their fusion provides complementary information. We conduct these analyses across three-, four-, and five-class sleep-stage tasks. We further evaluate PPG-based sleep staging using the MESA Sleep cohort to examine performance under a different pulse-signal acquisition setting and with a substantially larger cohort [5,6].

The main contributions of this study are as follows:

- We systematically compare handcrafted and STFT-based wearable signal representations across three-, four-, and five-class sleep-stage classification tasks.
- We quantify the contribution of individual and combined wearable modalities and examine how their contributions change with sleep-stage granularity.
- We evaluate whether handcrafted physiological features provide complementary information to learned STFT representations and compare alternative fusion strategies.
- We extend the pulse-based analysis to the larger MESA Sleep cohort to examine performance under a different PPG acquisition setting and training scale.

The paper is structured as follows. Section 2 reviews the related work on wearable sleep staging and signal representations. Section 3 describes the methodology, including the datasets, signal preprocessing, feature representations, and model architectures. Section 4 presents and discusses the experimental results. Finally, Section 5 summarizes the main findings and concludes the paper.

## 2. Related Work

### 2.1. Wearable Methods for Sleep Monitoring

Early wearable sleep-monitoring methods relied mainly on wrist actigraphy [7]. Actigraphy uses accelerometer-derived movement to estimate sleep and wake. It performs reasonably well for binary sleep and wake classification [8]. However, movement alone provides limited information for distinguishing REM from NREM sleep. Sundararajan et al. [9] used random forests with wrist accelerometry from more than 130 participants. Their results showed that multi-stage classification from movement alone remained challenging. Palotti et al. [10] reached a similar conclusion in a large, multi-cohort benchmark of sleep-wake classifiers, where machine-learning models trained on actigraphy performed well for wake detection but offered little multi-stage resolution. The addition of photoplethysmography (PPG) introduced cardiac information into wearable sleep staging [11]. PPG-derived heart rate and pulse-rate variability capture autonomic changes across sleep stages, reflecting differences in sympathetic and parasympathetic activity between NREM and REM sleep [11]. Walch et al. [12] combined PPG-derived heart rate with wrist accelerometry. The combined signals improved sleep-stage classification compared with accelerometry alone. Olsen et al. [13] later proposed a flexible deep learning architecture for temporal sleep-stage classification using accelerometry and PPG together, and reported consistent gains from combining the two signals across recording conditions. Motin et al. [14] likewise showed that multi-stage sleep classification was feasible from a PPG sensor alone, without accompanying accelerometry, though performance remained below that of multi-modal approaches. Sano et al. [15] also combined actigraphy, cardiac, and electrodermal activity (EDA) signals. Their results showed that multimodal physiological information improved sleep-stage classification. Boe et al. [16] reported a related finding using a wireless, multi-sensor wearable system, where combining physiological streams improved automated staging over single-sensor baselines. Later studies also reported complementary information from PPG and accelerometry, although performance varied across sleep stages [17]. A recent review confirmed the growing use of PPG and accelerometry for wearable sleep staging [4]. It also highlighted the limited ability of accelerometry alone to distinguish multiple sleep stages. However, large differences remain in sensor configurations, algorithms, and evaluation protocols across studies. These differences make direct comparisons difficult. Data scarcity compounds this problem, since PPG-labeled sleep data remain far less common than ECG data. Radha et al. [18] and Li et al. [19] both addressed this gap through transfer learning, pretraining on ECG-derived heart-rate information before fine-tuning on PPG, and reported improved staging accuracy relative to training on PPG alone. Signal quality is a further practical constraint: Orphanidou et al. [20] proposed signal-quality indices for PPG and ECG that are now commonly used to exclude unreliable wearable recordings before staging. EDA and skin temperature have received less attention. EDA shows stage-dependent changes during sleep, including characteristic activity during NREM sleep [3]. Herlan et al. [21] confirmed this pattern directly, showing that EDA activity differed systematically between sleep and wake and offered some value for sleep/wake classification specifically [21]. Skin temperature also changes with thermoregulatory activity across the sleep cycle. These signals may therefore provide information beyond cardiac and movement signals. More broadly, existing multimodal wearable studies often evaluate a fixed combination of sensors, whereas fewer systematically compare the incremental contributions of individual and combined modalities within the same evaluation framework.

### 2.2. Feature Representation and Deep Learning

Sleep-staging approaches broadly split along how much of the representation is hand-specified versus learned, though in practice the two ends of this spectrum share more structure than the split suggests. The classical paradigm treats staging as classification over hand-engineered features from 30 s epochs. For cardiac signals, this usually means time- and frequency-domain HRV or PRV statistics: mean and SD of interbeat intervals, RMSSD, low/high-frequency power ratios [22]. This feature set reflects a well-established autonomic pattern: HRV power shifts characteristically between REM and deep NREM sleep [22]. For accelerometry, this comprises activity counts, signal magnitude area, and axis-wise variance, borrowed from older actigraphy scoring [9]. EDA features come from skin-conductance level and the rate, amplitude, and duration of skin-conductance responses, following Sano et al.’s characterization of EDA during sleep [3]. Temperature features are simpler still, mostly level and slope, since thermoregulation moves slowly compared to cardiac or electrodermal signals. Modulation-spectrogram features are an alternative to raw signal or STFT, capturing slower amplitude-modulation patterns on top of a signal’s short-term spectrum [23]; Tallal et al. applied this to single-channel EEG from DREAMT and found it held up much better than STFT and wavelet baselines once participants were split by apnea severity [24], matching earlier work on modulation-spectrum robustness in other physiological and wearable tasks [25,26], and motivating modulation features as a next step for our BVP/ACC pipeline. Once extracted, these features are typically classified with either non-sequential models like random forests or sequence models that exploit predictable sleep-stage transitions. Topalidis et al. [27] used a random forest on HRV features derived from low-cost wearable ECG and PPG sensors, and additionally applied a random forest to flag poor-quality interbeat intervals before staging, showing that feature quality control matters as much as the classifier itself. Radha et al. [28] instead applied an LSTM directly to HRV features across a large, diverse dataset, and found that modeling long-term cardiac sleep architecture outperformed non-temporal and short-term recurrent classifiers. A consistent finding across this literature is that cardiac features (HR, PRV) carry the most useful information for REM and wake; accelerometry mainly helps with wake through movement [12], and EDA/temperature give smaller, less reliable gains that depend on what else is already in the feature set [21].

Deep learning approaches skip this handcrafted step and learn representations directly from raw or minimally processed physiological signal [29,30]. However, they inherit the same underlying design problem: how to capture local-epoch structure and inter-epoch transitions. They typically address this problem using related architectural building blocks (convolution for local shape, recurrence or attention for sequence) [31,32]. DeepSleepNet paired CNNs for shape-invariant EEG features with bidirectional LSTMs for epoch transitions, and matched or beat hand-engineered pipelines across datasets [33]. EEGNet took a more general route: a compact CNN using depthwise and separable convolutions, originally built for BCI tasks but now widely reused as a lightweight EEG backbone, including for sleep staging [34]. Eldele et al. proposed AttnSleep, combining a multi-resolution CNN with a multi-head attention encoder, and outperformed prior single-channel EEG models on three public datasets [35]. U-Time treated staging as dense segmentation with a U-Net-style encoder–decoder, and was more robust to hyperparameter changes than recurrent alternatives across seven PSG datasets [36]. SleepPPG-Net brought this end-to-end approach to wearables, running a residual plus temporal CNN directly on raw PPG for solid four-class staging [37]. Habib et al. also trained a CNN on raw PPG, achieving competitive accuracy without morphology features [38]. More recently, SleepMFormer replaced full self-attention with a task-driven, sparse Transformer encoder plus contrastive pretraining, cutting training and inference time by up to a third with no loss in accuracy on Sleep-EDF, PhysioNet, and SHHS [39]. NanoSleep takes a different angle: a Sinc-convolutional front end plus a gated dilated temporal CNN and CRF decoding, matching CNN–BiLSTM or transformer accuracy on single-channel EEG with far fewer parameters, useful for on-device staging [40].

These results shaped two choices here. First, we use an STFT representation of BVP and accelerometry magnitude with a U-Net-style spectral encoder, following U-Time [36] but more closely aligned with SleepPPG-Net’s wearable setting [37]. Second, for handcrafted features, we use a CNN–BiGRU, in DeepSleepNet’s spirit [33], to capture local patterns and inter-epoch transitions together. We then fuse the two branches, deep spectral and handcrafted recurrent, to test whether learned and physiologically grounded features add anything beyond either alone. Learned EEG/PPG representations [24,33,34,35,36,37,38,39,40] and handcrafted multimodal pipelines [12,15,17] have each been validated on their own datasets.

### 2.3. Generalization

A separate and increasingly emphasized concern is whether a trained model holds up outside its training distribution, across subjects, sensors, and clinical populations, rather than just on a held-out split of the same cohort. Some of this variability is addressable by adapting to a subgroup rather than training one model for everyone: Hossain and Kshirsagar fine-tuned a convolutional-recurrent model on demographic subgroups (age, sex, OSA severity) within a single 100-subject cohort, and found 35 of 37 subgroup-tuned models outperformed the pooled baseline, pointing to within-cohort population structure, not just architecture, as a real driver of accuracy [41]. Moreover, Refs. [24,42] showed apnea-based cohort classification for five-stage sleep classification. Van Der Aar et al. tested the complementary, cross-cohort question directly, comparing pretraining, training-from-scratch, and fine-tuning for single-channel EEG staging across eight target datasets; transfer learning gave little advantage over training from scratch in most age-matched cohorts, and only became clearly necessary in the smallest, suggesting that channel and population mismatch mainly hurt when combined with limited target data rather than independently [43]. Other work treats domain shift as a modeling problem to solve explicitly rather than data to fine-tune on. STDA-Net combines spectrogram representations with adversarial domain alignment to improve cross-dataset EEG staging without target-domain labels [44], and SleepDG aligns features at both the epoch and sequence level across multiple source datasets, reporting improved performance on domains never seen during training [45]. SleepPPG-Net2 applied the same multi-source idea to wearable PPG specifically, training across six datasets (2574 recordings) and improving cross-dataset generalization (Cohen’s kappa) by up to 21% compared with single-source training approaches [46].

Despite these advances, there is still limited work that examines wearable sleep staging from all of these perspectives within the same study. Most existing studies focus on a particular sensing modality, representation method, model architecture, or fixed multimodal configuration. In contrast, the present study systematically compares handcrafted and STFT-based representations, evaluates different combinations of wearable signals and hybrid fusion strategies, and investigates three-, four-, and five-class sleep-stage classification within a common subject-independent framework. This allows us to examine not only which approaches perform best, but also whether adding more physiological modalities or using more complex models consistently yields better-balanced sleep-stage classification.

## 3. Methods and Materials

**Figure 1:**
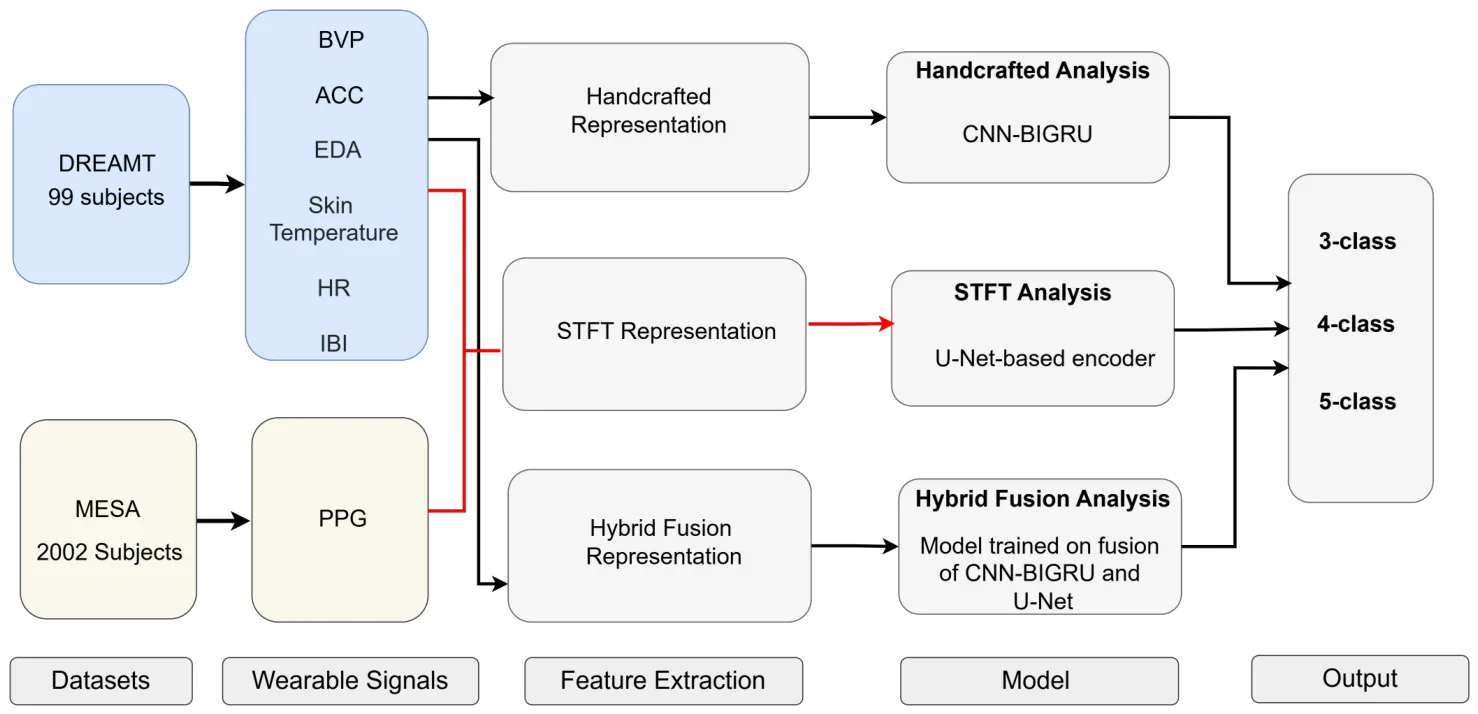
Overall study framework for multimodal wearable sleep-stage classification.

In this section, we describe the datasets, signal preprocessing, feature representations, model architectures, training procedure, and evaluation methods used in this study. The overall study design, including the DREAMT-based multimodal analysis and the independent MESA comparative analysis, is summarized in Figure 1.

### 3.1. Dataset

We used two sleep datasets: DREAMT [47], available through PhysioNet, and the Multi-Ethnic Study of Atherosclerosis (MESA) Sleep Ancillary Study, accessed through the National Sleep Research Resource (NSRR) [5,6]. DREAMT was used for the primary multimodal, feature-representation, and fusion experiments, while MESA was used to evaluate pulse-based sleep-stage classification in a larger cohort and under a different acquisition setting. Participant demographics and sleep characteristics are summarized in Table 1.

#### 3.1.1. DREAMT Dataset

DREAMT (Dataset for Real-time Sleep Stage Estimation using Multisensor Wearable Technology) [47] is a publicly available dataset containing synchronized wrist-worn physiological signals from 100 adults undergoing overnight clinical sleep assessment. Signals were recorded using the Empatica E4 wristband and include blood volume pulse (BVP), triaxial accelerometry (ACC), electrodermal activity (EDA), skin temperature, heart rate (HR), and interbeat interval (IBI), together with PSG-derived sleep-stage annotations assigned by expert sleep technicians. The multimodal recordings were used to investigate the individual and combined contributions of wearable modalities and to compare handcrafted, STFT-based, and hybrid feature representations. After applying the signal-quality criteria, 99 participants were retained for analysis.

#### 3.1.2. MESA Sleep Dataset

The MESA Sleep Ancillary Study was accessed through the NSRR following approval of the required Data Access and Use Agreement [5,6]. The dataset contains overnight in-home polysomnography recordings from 2056 participants. Although multiple physiological signals are available, only the photoplethysmographic (PPG) waveform was used in this study to provide a pulse-based evaluation complementary to the wrist BVP available in DREAMT. The PPG signal is provided as the Pleth channel and was recorded from the finger using a pulse-oximetry sensor at 256 Hz. After applying the inclusion and signal-quality criteria, 2002 participants were retained for analysis. Two MESA cohorts were evaluated: a 100-participant subset (MESA-100) and the complete eligible cohort (MESA-all; n=2002). MESA-100 provided a cohort size comparable to DREAMT, while MESA-all enabled evaluation using a substantially larger training population. The construction of MESA-100 is described in Section 3.3. MESA was analyzed independently as an additional cohort rather than as a direct cross-dataset external validation of models trained on DREAMT.

#### 3.1.3. Participant Demographics and Sleep Parameters

Participant demographic and sleep characteristics for DREAMT, MESA-100, and MESA-all are summarized in Table 1. In addition to these cohort differences, DREAMT and MESA also differ in data-acquisition conditions, including pulse-signal sensor placement, sampling configuration, and the set of available wearable modalities. These acquisition differences may also contribute to the observed performance differences between the datasets.

### 3.2. Sleep-Stage Definitions and Label Harmonization

Both datasets provide sleep-stage labels for consecutive 30 s epochs. The labels include Wake, N1, N2, N3, and REM. We excluded epochs labeled as movement, preparation, unknown, or unscored. We evaluated three sleep-stage classification tasks. Table 2 summarizes the label mappings. For the five-class task, we retained the original five stages. For the four-class task, we combined N1 and N2 as Light sleep and retained N3 as Deep sleep. For the three-class task, we combined N1, N2, and N3 as NREM sleep. We applied the same label mappings to both datasets.

### 3.3. Data Quality Control and Cohort Selection

We screened both datasets for invalid signals, missing sleep-stage labels, recording discontinuities, and alignment with the 30 s scoring grid. We excluded invalid epochs and processed continuous recording segments separately to avoid crossing signal gaps. We did not impute missing sleep-stage labels. For DREAMT, retained epochs were required to contain a complete 30 s interval with a single valid sleep-stage label. Signal-integrity checks verified finite BVP and ACC values, timestamp continuity, and valid ACC sampling structure. One participant was excluded because of accelerometer failure, leaving 99 participants, 102 continuous sequences, and 79,468 valid epochs. Table 3 summarizes the resulting class distribution, showing that the five-class distribution is imbalanced, with N3 being the least represented stage and N1 also occurring less frequently than Wake and N2. We considered this class distribution when selecting balanced evaluation metrics for the study. For MESA, PPG recordings were audited for signal and annotation quality. Recordings were flagged for EDF–annotation duration mismatches greater than 1 s, non-finite samples, more than 50% identical adjacent samples, flatlines longer than 10 s, PPG interquartile range below $10^{-4}$ of the physical signal range, saturation affecting more than 5% of samples, or pulse-band power ratio below 0.05. Audit flags were not automatic subject-level exclusion criteria; we retained usable portions as independent continuous sequences. Participants were included when at least 780 usable epochs (6.5 h) were available and at least one continuous sequence contained 360 epochs (3 h). After quality control, 2002 participants remained. We constructed MESA-100 by selecting 25 participants from each AHI category (normal, mild, moderate, and severe) to provide a cohort size comparable to DREAMT, and balancing the subset by sex. We used a fixed random seed of 2022 for reproducible sampling.

### 3.4. Signal Preprocessing

We processed each continuous recording sequence independently and aligned the signals with the 30 s sleep-stage annotations. DREAMT used separate preprocessing pipelines for the STFT and handcrafted representations, while MESA used PPG for STFT-based classification.

#### 3.4.1. DREAMT Signal Preprocessing

For the STFT representation [48,49], we processed BVP and ACC following [13]. We low-pass filtered BVP at 12 Hz using a fourth-order zero-phase Chebyshev Type-I filter and downsampled it from 64 to 32 Hz. We then applied a fifth-order zero-phase Butterworth band-pass filter between 0.1 and 8 Hz. Finally, we applied a centered 300 s adaptive IQR normalization and clipped the normalized signal to [−20,20]. We restored ACC to its native 32 Hz sampling rate and normalized each axis using participant-specific IQR statistics. The processed BVP and ACC signals were then used to generate the STFT representations. For handcrafted features, we retained BVP at 64 Hz and applied a fourth-order zero-phase Butterworth band-pass filter between 0.5 and 8 Hz before pulse detection. We restored ACC, EDA, skin temperature, and HR to their native sampling rates. We decomposed EDA into tonic and phasic components using a 0.05 Hz low-pass filter. We derived interbeat intervals from consecutive BVP peaks and retained intervals between 0.30 and 2.00 s for PRV analysis. All processing remained within continuous recording sequences.

#### 3.4.2. MESA Signal Preprocessing

We processed the MESA finger PPG using the same pulse-signal pipeline used for the DREAMT STFT representation. We low-pass filtered the 256 Hz PPG at 12 Hz, downsampled it to 32 Hz, and applied a 0.1–8 Hz Butterworth band-pass filter. We then applied the centered 300 s adaptive IQR normalization and clipped the signal to [−20,20]. We used the resulting PPG signal to generate the STFT.

### 3.5. Feature Representation and Extraction

We generated two complementary representations: STFT-based time–frequency representations and handcrafted physiological features. We aligned both representations with the corresponding 30 s sleep-stage epochs and generated them independently within each continuous recording sequence.

#### 3.5.1. STFT-Based Time–Frequency Representation

Following [13], to maintain methodological consistency with an established wearable sleep-staging framework, we computed the STFT continuously within each valid recording sequence rather than independently for each 30 s epoch. We used a 10 s Blackman window with 9 s overlap, producing one spectral frame per second. Each sleep-stage epoch therefore corresponded to 30 consecutive spectral frames. For BVP and PPG, we used a 512-point Fourier transform and retained 64 frequency bins below 4 Hz. We converted spectral power to the natural logarithmic scale. We applied the same STFT parameters to ACC. For ACC, we evaluated three representations: axis-averaged, magnitude, and separate triaxial spectrograms. For the magnitude representation, we computed

(1) $$a_{\mathrm{mag}}\left(t\right)=\sqrt{a_{x}{\left(t\right)}^{2}+a_{y}{\left(t\right)}^{2}+a_{z}{\left(t\right)}^{2}}.$$

**Figure 2:**
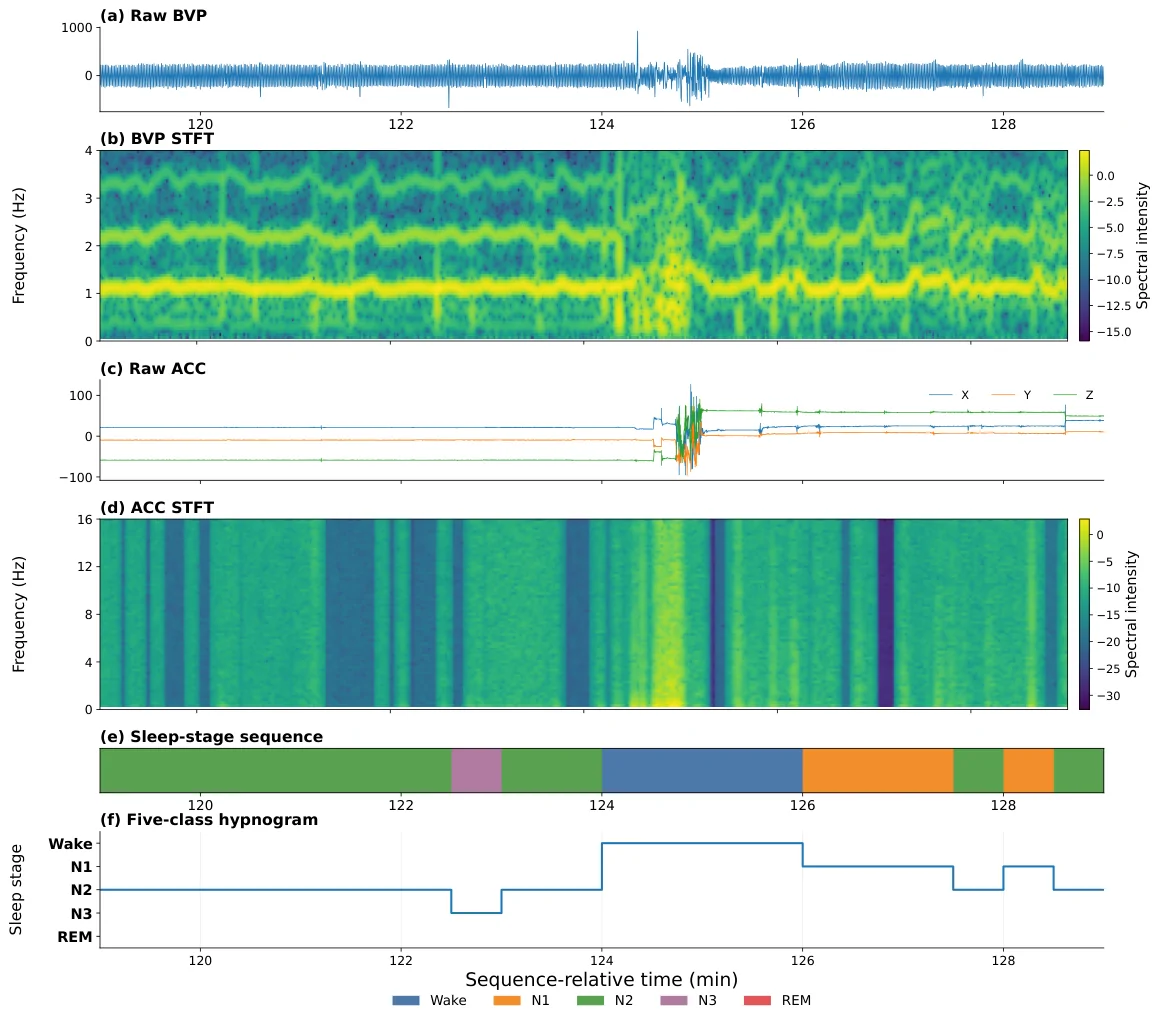
Representative raw BVP and ACC signals, corresponding STFT representations, and aligned five-class sleep-stage sequence from a DREAMT recording.

For the axis-averaged representation, we averaged the three axis-specific spectrograms element-wise. For the triaxial representation, we retained the three spectrograms as separate input channels. During model training, we used sequences of up to 1024 consecutive epochs (approximately 8.5 h). We zero-padded shorter sequences and masked the padded positions during training and evaluation. To illustrate the resulting time–frequency representations, Figure 2 shows a representative DREAMT segment containing the raw BVP and ACC signals, their corresponding STFT representations, and the aligned five-class sleep-stage sequence. The example demonstrates how changes in the physiological signals and their spectral content correspond temporally with transitions between sleep stages.

#### 3.5.2. Handcrafted-Feature Extraction

We extracted 112 handcrafted features from DREAMT to characterize cardiac, movement, electrodermal, and skin-temperature activity. Most features were computed within each 30 s epoch. PRV and selected temporal features used neighboring intervals within the same continuous recording sequence and did not cross masked epochs or recording gaps. The feature bank contained 37 cardiac, 41 ACC, 20 EDA, and 14 skin-temperature features (Table 4). Cardiac features described BVP morphology, interbeat intervals, HR, and PRV [50]. ACC features characterized movement intensity, orientation, temporal changes, and frequency content [51]. EDA features captured tonic and phasic activity [52], while temperature features described level, variability, trend, and temporal changes [8]. The complete feature definitions are provided in the Abbreviations.

#### 3.5.3. Feature Assembly and Preprocessing

We aligned modality-specific features by participant, sequence, and epoch and combined them into a 112-dimensional feature vector. Pairwise Spearman correlation identified 59 feature pairs with |ρ|≥0.95, all within individual modalities. We retained all features because the analysis showed no highly correlated cross-modality pairs and the overall dimensionality remained moderate. We fitted missing-value imputation and feature scaling using only the training participants within each cross-validation fold. We then applied the fitted transformations unchanged to the validation and test participants.

### 3.6. Model Architectures

This section describes the model architectures evaluated in this study, including the STFT-based U-Net, the handcrafted-feature model, and the proposed fusion model.

#### 3.6.1. STFT-Based U-Net Model

The STFT-based sleep-stage classifier was implemented following the U-Net-inspired architecture proposed by [13]. The baseline configuration used two modalities: BVP and ACC. We combined the spectrograms of these two modalities into two input channels and processed them together with a temporal encoder–decoder network. The input modalities contained 1024 consecutive 30 s epochs, with each represented by 30 one-second spectral frames and 64 frequency bins. The encoder gradually reduced the temporal and frequency dimensions while increasing the number of effectively learned feature maps. The decoder used skip connections to restore temporal resolution and combine features from the corresponding encoder level. The implementation used 10 encoder–decoder levels, 16 initial filters, a convolutional kernel of size 16×3, and a temporal frequency reduction factor of 2×2. This model served as both the STFT-based baseline and the spectral feature extractor for the proposed fusion framework.

#### 3.6.2. Handcrafted-Feature Temporal Model

To learn temporal patterns from handcrafted features, we used a temporal CNN–BiGRU model. We processed handcrafted features from consecutive valid 30 s epochs as continuous sequences from the same recording. Context windows were restricted to each participant’s continuous valid recording sequence and did not extend across recording gaps or masked epochs. To handle sequence boundaries, we padded the context using only values from the same valid sequence. To capture short-term patterns between neighboring epochs, the input feature sequence was first processed by a temporal convolutional encoder with 64 output channels. The generated representation was then passed through a bidirectional gated recurrent unit with 64 hidden units in each direction. The forward and backward hidden states were combined to form a 128-dimensional embedding for each target epoch. We then applied a dropout rate of 0.25 to reduce overfitting before passing the embedding to the final fully connected classification layer.

#### 3.6.3. Proposed Dual-Branch STFT–Handcrafted Fusion Model

**Figure 3:**
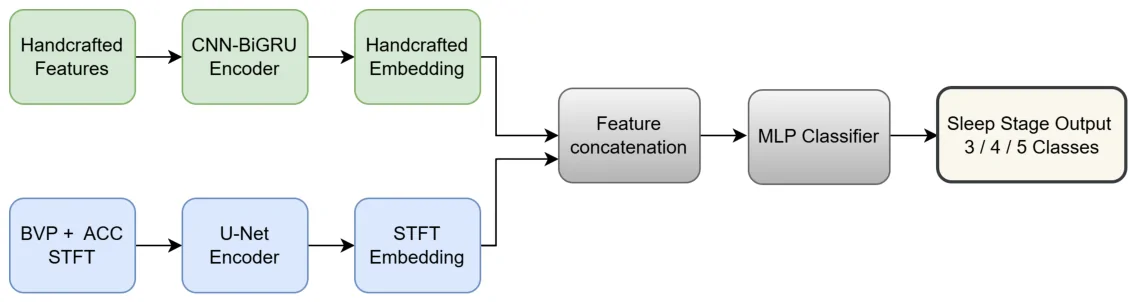
Hybrid fusion architecture for wearable sleep-stage classification.

The proposed fusion model integrates complementary information from STFT-based and handcrafted feature representations and assesses the contribution of multimodal fusion over single-modality configurations. The STFT branch learns time–frequency patterns directly from the signals and captures longer-term sleep-stage dependencies [13], whereas the handcrafted branch encodes physiologically motivated morphological, statistical, temporal, and frequency-domain characteristics. These two representations therefore provide complementary views of the wearable signals, with one learned from the time–frequency structure and the other guided by established physiological descriptors. Figure 3 shows the architecture. We trained the two branches separately and used the checkpoints that performed best on the validation set to generate an embedding for each valid 30 s epoch. We then aligned the STFT and handcrafted embeddings using the participant ID, recording sequence ID, and the epoch position within the sequence. We included an epoch in the fusion dataset only when these identifiers and the corresponding sleep-stage label matched across both branches. For epoch *i*, let $z_{i}^{\mathrm{STFT}}$ denote the embedding generated by the STFT branch and $z_{i}^{\mathrm{HC}}$ denote the embedding generated by the handcrafted-feature branch. The aligned STFT and handcrafted embeddings were concatenated to form a joint feature representation [53]:

(2) $$z_{i}=\left[z_{i}^{\mathrm{STFT}}\;∥\;z_{i}^{\mathrm{HC}}\right],$$

where ‖ denotes feature-wise concatenation.

(3) $${\tilde{z}}_{i}=\frac{z_{i}-\mu_{\mathrm{train}}}{\sigma_{\mathrm{train}}},$$

where $\mu_{\mathrm{train}}$ and $\sigma_{\mathrm{train}}$ denote the feature-wise mean and standard deviation estimated from the training partition [54]. The validation and test sets used the same scaling parameters. During fusion training, the STFT and handcrafted encoders remained frozen, and only the multilayer perceptron (MLP) classifier was updated. The fusion MLP first applied layer normalization [55], followed by a fully connected layer with 128 units, GELU activation [56], and dropout. A second fully connected layer reduced the embedding to 64 dimensions, followed again by GELU activation and dropout. The final linear layer produced one logit for each sleep-stage class. The resulting logit vector can be expressed as

(4) $$o_{i}=f_{\mathrm{MLP}}\left({\tilde{z}}_{i}\right),$$

where $f_{\mathrm{MLP}}\left(\cdot \right)$ represents the fusion classifier and $o_{i}\in R^{C}$ contains the logits for the *C* sleep-stage classes. During training, these logits were passed directly to the weighted cross-entropy loss. During inference, softmax was used to convert the logits into class probabilities [57]:

(5) $$p_{i,c}=\frac{\exp(o_{i,c})}{\sum_{k=1}^{C}\exp\left(o_{i,k}\right)},$$

where $p_{i,c}$ is the predicted probability that epoch *i* belongs to class *c*. The final predicted sleep stage was then obtained as

(6) $${\hat{y}}_{i}=\arg\max_{c}p_{i,c}.$$

### 3.7. Model Training and Selection

The training configuration of the individual branches and the proposed fusion model is summarized in Table 5. The dataset was split at the participant level to prevent data leakage, with all recordings and sequences from the same participant assigned to one partition. We reserved 15 participants as an independent holdout test set and used the remaining participants for five-fold cross-validation. In each fold, approximately 68–69% of the development participants were used for training, 12% for validation, and 19–20% for cross-validation testing. To improve reproducibility, all randomized procedures used a fixed seed of 2022.

We estimated all preprocessing parameters only from the training partition. To reduce the effect of sleep-stage imbalance, we trained all models using effective-number-weighted cross-entropy loss with β=0.999. The validation set was used for model selection and early stopping, whereas the cross-validation test set was used for performance comparison. After the final model configuration was fixed, the holdout set was evaluated once to estimate performance on unseen participants.

### 3.8. Experimental Setup and Reproducibility

We implemented all experiments in Python 3.9.21 using PyTorch 2.8.0 with CUDA 12.8. For model training, we used GPU resources of the ACCESS Delta high-performance computing system, with NVIDIA A40 GPUs (NVIDIA Corporation, Santa Clara, CA, USA) [58,59]. Additional preprocessing and experimental checks were performed on a local workstation equipped with an NVIDIA Quadro RTX 6000 GPU with 24 GB memory. Throughout the experiment, a fixed seed of 2022 was used, and the same participant-level data splits were maintained across the compared models to support reproducibility and fair comparison.

### 3.9. Evaluation Metrics

We evaluated sleep-stage classification at the 30 s epoch level using accuracy, Macro-F1 [60], Cohen’s κ, and class-specific F1-score [61]. We also examined confusion matrices to identify common misclassifications between sleep stages. We used Macro-F1 as the primary model-selection metric because it gives equal weight to each class and is less affected by class imbalance than overall accuracy. However, Macro-F1 can decrease when performance on minority classes worsens, even if overall accuracy increases. Therefore, a higher accuracy together with a lower Macro-F1 may indicate greater emphasis on the more frequent classes at the expense of balanced performance across sleep stages.

Accuracy measures the proportion of correctly classified epochs [62]. Macro-F1 is the unweighted mean of the class-specific F1-scores and was calculated as

(7) $$\mathrm{Macro}-F1=\frac{1}{C}\sum_{c=1}^{C}F1_{c},$$

where *C* is the number of sleep-stage classes. Cohen’s κ measures agreement between predicted and reference labels while accounting for agreement expected by chance:

(8) $$\kappa =\frac{p_{o}-p_{e}}{1-p_{e}},$$

where $p_{o}$ and $p_{e}$ denote the observed and expected agreement, respectively [63].

For cross-validation, we evaluated the validation-selected checkpoint on the test participants of each fold and reported the mean ± standard deviation across five folds. After fixing the final model configuration, we evaluated it once on the independent holdout set and reported those results separately. We excluded masked and padded epochs from all metric calculations. For key representation and fusion comparisons, two-sided paired *t*-tests were performed on fold-wise Macro-F1 values from the same five subject-independent cross-validation folds, with p<0.05 considered statistically significant.

## 4. Results and Discussion

In this section, we present the experimental results and discuss the main findings in relation to the study objectives and existing literature.

### 4.1. Accelerometry Representation Analysis

The objective of this experiment was to determine whether wrist accelerometry representation affects sleep-stage classification. We specifically examined whether preserving directional information from the three accelerometer axes provides an advantage over simpler representations of overall movement. We compared three STFT-based ACC representations: axis-averaged, magnitude-based, and separate triaxial representations. We generated all three representations using the same preprocessing and STFT parameters and evaluated them with the same model configuration. This controlled comparison allowed us to isolate the effect of ACC representation on classification performance.

Table 6 demonstrates the performance of the three ACC representations for the four-class sleep-staging tasks. ACC magnitude achieved the highest Macro-F1 of 0.4248 and Cohen’s κ of 0.2997. Retaining the three axes separately did not improve class-balanced performance (Macro-F1 = 0.4096), while averaging the axis spectra achieved a Macro-F1 of 0.4185. These results indicate that preserving axis-specific directional information did not provide an additional advantage for sleep-stage classification in this setting. ACC magnitude was therefore selected for the subsequent experiments because it provided the strongest overall class-balanced performance while offering a compact representation that is less dependent on device orientation.

### 4.2. Architecture Ablation for Handcrafted Features

This experiment aimed to select an appropriate architecture for the handcrafted feature representation. We compared conventional machine-learning and temporal deep learning models using the same 112-dimensional feature set and subject-independent five-fold evaluation protocol. As shown in Table 7, Random Forest and RF–HMM achieved the highest overall accuracy, with RF–HMM reaching 0.6647. However, their Macro-F1 and Cohen’s κ were substantially lower than those of the temporal deep learning models. CNN–BiGRU achieved the highest Macro-F1 (0.4251) and Cohen’s κ (0.3475), while maintaining a comparable accuracy of 0.6612. These results show that CNN–BiGRU provided the best class-balanced performance among the evaluated architectures. Its combination of local feature extraction and temporal modeling may help capture patterns across successive sleep epochs. This also suggests that temporal modeling is more important for this task than relying on additional non-temporal classifiers. We therefore selected CNN–BiGRU for the subsequent handcrafted-feature experiments.

### 4.3. Feature Representation Comparison

This experiment aimed to determine how feature representation affects wearable sleep-stage classification. We compared handcrafted, STFT-based, and hybrid representations using the same BVP and ACC signals across the 3-, 4-, and 5-class tasks. Table 8 shows that STFT provided better class-balanced performance than handcrafted features. For the 3-class task, STFT increased accuracy from 68.16% to 73.65%, Cohen’s κ from 0.3271 to 0.4788, and Macro-F1 from 0.5057 to 0.6404. For the 4- and 5-class tasks, handcrafted features achieved slightly higher accuracy than STFT, but STFT produced substantially higher Cohen’s κ and Macro-F1. In the 5-class task, for example, Macro-F1 increased from 0.3366 to 0.4376 despite a small decrease in accuracy. The improvement in Macro-F1 suggests that the STFT representation preserves discriminative time–frequency information that manually designed features may not fully capture. This observation is consistent with previous studies showing that time–frequency representations and data-driven feature learning can provide effective representations for automatic sleep-stage classification [64,65,66]. The hybrid representation achieved the best overall performance across all three tasks. Compared with STFT alone, fusion increased Macro-F1 from 0.6404 to 0.6476 for the 3-class task, from 0.5083 to 0.5108 for the 4-class task, and from 0.4376 to 0.4464 for the 5-class task. These modest gains suggest that STFT already captures most of the discriminative information in BVP and ACC, while handcrafted features provide additional complementary information.

Overall, performance decreased as the number of classes increased, reflecting the greater difficulty of distinguishing individual sleep stages. Similar challenges in separating fine-grained sleep stages have been reported in previous wearable sleep-staging studies [67,68,69]. These results support STFT as the primary representation for BVP and ACC, while hybrid fusion provides a small but consistent additional benefit.

### 4.4. Handcrafted Modality Contribution Across Sleep-Stage Granularities

This analysis aimed to determine which wearable modalities provide the most useful handcrafted features and whether combining additional modalities improves sleep-stage classification. We evaluated individual and multimodal combinations of BVP, PRV, HR, ACC, EDA, and temperature across the 3-, 4-, and 5-class tasks. Table 9, Table 10 and Table 11 show that adding more modalities did not consistently improve class-balanced performance. Among the individual modalities, HR achieved the highest Macro-F1 for both the 3-class (0.6011) and 4-class (0.4510) tasks. HR also provided the strongest REM classification, with F1 scores of 0.4315 and 0.4174, respectively. In contrast, ACC was particularly informative for Wake, indicating that cardiac and movement features capture complementary aspects of sleep physiology. The 5-class task was more challenging. The combination of BVP, PRV, HR, ACC, and EDA achieved the highest Macro-F1 of 0.3679 and Cohen’s κ of 0.3392. However, N1 and N3 remained substantially more difficult to classify than Wake, N2, and REM. This difficulty may reflect both their lower representation and their physiological similarity to neighboring sleep stages.

Overall, the handcrafted analysis shows that different modalities contribute to different sleep stages. Cardiac features are particularly useful for REM, while ACC provides complementary information for Wake. Importantly, adding more modalities does not necessarily improve performance, indicating that the relevance of the selected physiological signals is more important than the number of modalities.

The class-specific results further show that performance varied considerably across sleep stages. NREM in the 3-class task and Light sleep in the 4-class task achieved consistently high F1 scores across most feature configurations. For example, NREM F1 remained around 0.75–0.80 in the 3-class task. Their stronger performance may partly reflect their greater representation in the dataset, which provides more training examples, rather than physiological separability alone.

The differences became more pronounced in the 5-class task. BVP + PRV + HR + ACC + EDA achieved the highest overall Macro-F1 of 0.3679 and Cohen’s κ of 0.3392, but N1 and N3 remained difficult to classify. In contrast, N2 showed consistently stronger performance. This pattern may reflect both class imbalance and the physiological similarity of N1 and N3 to neighboring stages. Overall, the results show that each wearable modality contributes differently across sleep stages. Cardiac features were particularly informative for REM, while ACC contributed more strongly to Wake detection. Thus, modality selection should consider stage-specific information rather than simply increasing the number of physiological signals.

### 4.5. STFT-Based Modality Contribution Across Sleep-Stage Granularities

This analysis aimed to determine the individual and complementary contributions of BVP and ACC in the time–frequency domain. We compared BVP alone, ACC magnitude alone, and their combination across the 3-, 4-, and 5-class sleep-stage tasks. Table 12, Table 13 and Table 14 show that BVP was consistently more informative than ACC alone. In the 3-class task, BVP achieved a Macro-F1 of 0.6255, compared with 0.4817 for ACC. Combining both modalities further increased Macro-F1 to 0.6404. The largest modality difference occurred for REM, where BVP achieved an F1-score of 0.4442 compared with only 0.0758 for ACC. The combined model slightly improved REM F1 to 0.4631. A similar pattern appeared in the 4-class task. BVP + ACC achieved the highest Macro-F1 of 0.5083, compared with 0.4996 for BVP and 0.4248 for ACC. However, BVP alone remained slightly better for REM (0.4543 versus 0.4493). These results suggest that ACC provides complementary movement information, particularly for Wake and NREM discrimination, whereas BVP carries stronger information for REM.

The pattern changed in the 5-class task. As shown in Table 14, BVP alone achieved the highest Macro-F1 of 0.4520, compared with 0.4376 for BVP + ACC and 0.3731 for ACC. BVP also performed best for N2, N3, and REM, while adding ACC produced only small improvements for Wake and N1. Thus, the benefit of accelerometry decreased when NREM was divided into N1, N2, and N3. The stronger contribution of BVP, particularly for REM, is consistent with the autonomic cardiovascular changes associated with REM sleep [22]. Changes in heart rate and beat-to-beat variability alter the peripheral pulse waveform, and the STFT representation can capture their temporal and spectral dynamics. In contrast, ACC primarily reflects body movement and may provide less stage-specific information during sleep.

Overall, BVP was the stronger individual STFT modality, while ACC provided complementary information mainly in the 3- and 4-class tasks. The results also show that combining modalities does not always improve performance as sleep-stage classification becomes more fine-grained. Previous studies have reported benefits from ECG-to-PPG transfer learning [18,19]; therefore, cardiovascular pretraining may provide an avenue to further improve the BVP representation.

### 4.6. Fusion of STFT and Handcrafted Feature Representations

The objective of this experiment was to determine whether handcrafted features provide complementary information beyond the BVP–ACC STFT representation. We fused the STFT model with handcrafted features from individual and combined modalities and evaluated the resulting models across the 3-, 4-, and 5-class tasks. Table 15, Table 16 and Table 17 show modest but consistent benefits from selected fusion configurations. For the 3-class task, fusing handcrafted BVP + ACC with STFT produced the best performance, increasing Macro-F1 from 0.6404 to 0.6476 and accuracy from 73.65% to 75.17%. For the 4-class task, BVP + PRV + HR + Temperature achieved the highest Macro-F1 of 0.5178, compared with 0.5083 for STFT alone. In the 5-class task, BVP + ACC again produced the highest Macro-F1, increasing it from 0.4376 to 0.4464.

These results indicate that handcrafted features provide complementary information, but their contribution depends on the classification task. Importantly, adding more modalities did not consistently improve Macro-F1. For example, the full multimodal combination achieved the highest accuracy and Cohen’s κ in the 4-class task, but not the highest Macro-F1. This pattern suggests that the additional modalities improved performance for the more frequent classes without producing equally balanced gains across all sleep stages. EDA and temperature showed smaller and less consistent contributions. However, temperature was useful in the 4-class task, where BVP + PRV + HR + Temperature achieved the highest Macro-F1. This supports previous studies showing that EDA and peripheral temperature contain sleep-related physiological information [3,16,21], although their contribution appears to be task-dependent.

Overall, STFT captured most of the discriminative information available from BVP and ACC, while handcrafted features provided a small complementary benefit. The best fusion configuration changed with sleep-stage granularity, showing that adding more modalities is not necessarily more effective than selecting physiologically relevant features for the target task. Paired fold-wise analysis further showed that the STFT BVP + ACC representation significantly outperformed both the corresponding handcrafted BVP + ACC representation (t(4)=9.02, p<0.001) and the best-performing handcrafted single modality, HR (t(4)=3.60, p=0.0227). In contrast, the numerical gains obtained through frozen score-level fusion were not statistically significant. For BVP + ACC, Macro-F1 increased from 0.5083 to 0.5108 (t(4)=2.06, p=0.108), while the best score-level fusion configuration reached 0.5178 (t(4)=1.66, p=0.173). These findings support the advantage of STFT over handcrafted representations while indicating that the relatively small fusion gains should be interpreted cautiously.

**Figure 4:**
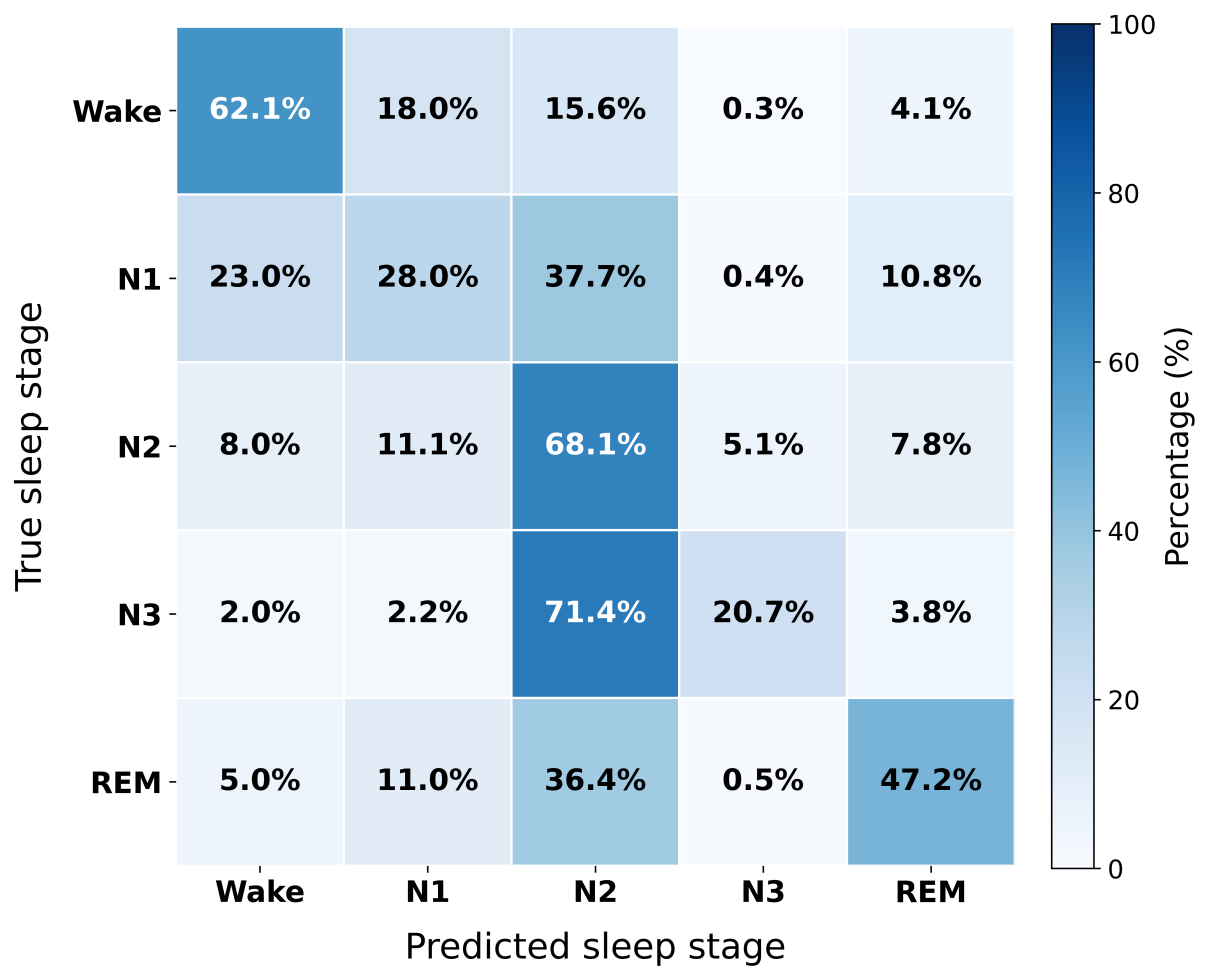
Row-normalized confusion matrix for the five-class BVP + ACC fusion model.

To further examine the error pattern of the final five-class BVP + ACC fusion model, Figure 4 shows the row-normalized confusion matrix obtained from pooled out-of-fold test predictions. N2 and Wake were classified more reliably, while N1 and N3 remained more difficult and were frequently confused with N2.

### 4.7. Fusion Strategy Ablation

This experiment aimed to determine whether adaptive fusion could improve on simple frozen concatenation. We compared three strategies: frozen concatenation, joint fine-tuning of both encoders, and gated residual fusion. For gated fusion, the STFT and handcrafted embeddings were projected into a common 64-dimensional space, and the handcrafted contribution was controlled by a learned sigmoid gate:

$$z_{\mathrm{fusion}}=s+g⊙h,$$

where *s* and *h* are the projected STFT and handcrafted embeddings, respectively, and *g* is the learned gating coefficient. As shown in Table 18, frozen concatenation achieved the highest Macro-F1 of 0.5144±0.0337. Joint fine-tuning and gated fusion increased accuracy to 0.6949 and 0.6940, respectively, but reduced Macro-F1 to 0.4908 and 0.4993. The gated model learned a mean coefficient of 0.477±0.073, indicating that it continued to incorporate the handcrafted representation. Overall, increasing fusion complexity did not improve class-balanced performance. Although the adaptive strategies achieved higher accuracy, frozen concatenation produced the highest Macro-F1 and comparable Cohen’s κ. We therefore retained frozen concatenation as the final fusion strategy.

### 4.8. MESA Evaluation: Effect of Acquisition Setting and Cohort Size

The objective of the MESA evaluation was to examine whether pulse-based sleep-stage classification remained effective under a different acquisition setting and whether increasing the training cohort improved performance. We first evaluated MESA-100, a 100-subject subset selected to provide a cohort more comparable in size and apnea-severity distribution to DREAMT. We then trained the same approach on the full MESA cohort to assess the benefit of a larger, more diverse training population.

#### 4.8.1. MESA-100 Comparison

MESA-100 achieved stronger class-balanced performance than the corresponding DREAMT pulse-based experiments across the 3-, 4-, and 5-class tasks. This result suggests that the acquisition setting can influence the sleep-related information captured by peripheral pulse signals. DREAMT acquired BVP from the wrist, whereas MESA acquired PPG using a finger pulse oximeter. However, this comparison should not be interpreted as evidence that finger PPG is inherently superior to wrist BVP. The datasets also differ in acquisition hardware, original sampling rate, cohort characteristics, and other recording conditions. Therefore, the MESA-100 results indicate that acquisition conditions may influence performance, but this comparison cannot isolate the contribution of individual factors.

#### 4.8.2. Effect of Cohort Size

We next compared MESA-100 with the full MESA cohort to examine the effect of increasing the training population. As shown in Table 19, performance improved consistently across all three classification tasks. Macro-F1 increased from 0.7059 to 0.8430 for the 3-class task, from 0.6564 to 0.7101 for the 4-class task, and from 0.5515 to 0.6265 for the 5-class task. Cohen’s κ showed the same overall trend.

These results demonstrate a clear benefit from increasing the number and diversity of subjects available for training. Nevertheless, performance still decreased as the classification task became more fine-grained. Thus, additional training data improved the robustness of PPG-based sleep staging but did not eliminate the difficulty of distinguishing individual sleep stages.

#### 4.8.3. Stage-Specific Performance and Cross-Dataset Limitations

**Figure 5:**
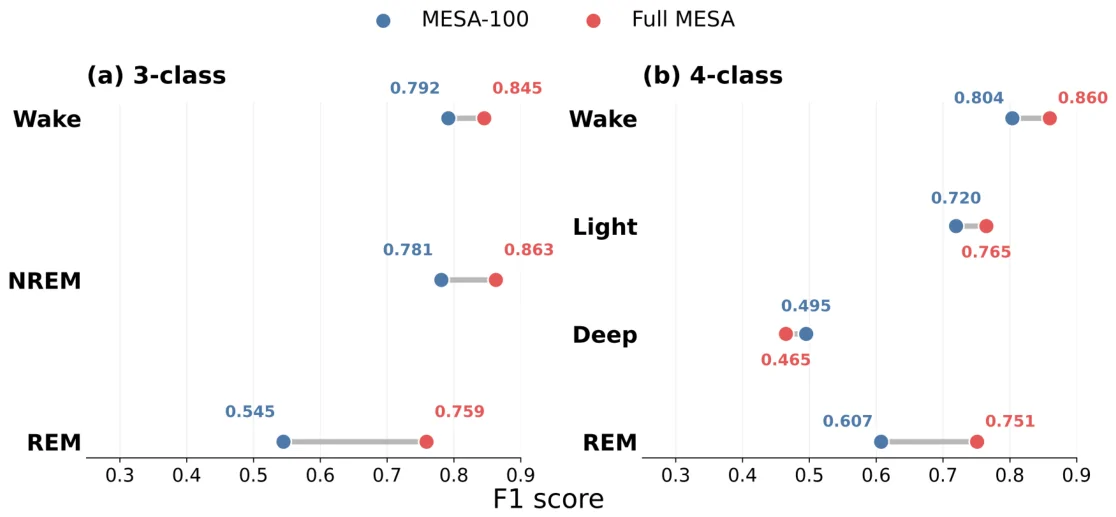
Stage-wise F1 comparison between the MESA-100 and Full MESA cohorts for three- and four-class sleep-stage classification.

The class-specific results provide further insight into how cohort size affects sleep-stage performance. Figure 5 compares stage-wise F1 scores for the three- and four-class settings. In general, the Full MESA cohort improved performance across most stages, although the magnitude of improvement varied by stage. In the four-class setting, Deep sleep showed a slight decrease despite the larger training cohort.

In the 5-class task, the corresponding stage-wise F1 scores are reported in Table 20. Wake, N2, and REM reached F1 scores of 0.8622, 0.6811, and 0.7443 with the full cohort. N1 and N3 remained considerably lower at 0.3624 and 0.4823, respectively.

The persistent difficulty of N1 and N3 is particularly important because a similar pattern was observed in DREAMT. Their lower performance therefore persisted across different cohorts, pulse-acquisition locations, and training-set sizes. Although class imbalance may contribute to this difficulty, it is unlikely to fully explain it. N1 is a transitional stage with overlapping characteristics and potentially greater annotation uncertainty, while epoch-level signal artifacts may further affect wearable measurements. In addition, the electrophysiological features used to define NREM stages in PSG may not be fully represented in peripheral cardiovascular signals. Overall, the MESA experiments provide two main findings. First, the stronger MESA-100 results suggest that pulse acquisition and other dataset-specific conditions can influence sleep-stage classification performance. Second, increasing the training population substantially improves PPG-based classification, but does not fully resolve fine-grained NREM staging. The consistent difficulty of N1 and N3 across DREAMT and MESA therefore suggests a broader limitation of peripheral wearable signals for reproducing the distinctions between neighboring NREM stages.

### 4.9. Limitations

This study has several limitations. First, we did not perform direct cross-dataset generalization by training on DREAMT and testing the same model on MESA or another independently collected dataset. This remains an important direction for future work. Second, the analysis was based on existing research datasets and did not include real-world consumer wearable recordings or performance stratification by clinical subgroups such as OSA severity. Third, N1 and N3 showed lower classification performance than the other sleep stages, which may be related to class imbalance, stage-transition ambiguity, and the limited information available from peripheral wearable signals. Finally, the current study evaluated STFT as the main time–frequency representation and did not compare it with other multi-resolution methods. Future work will investigate alternatives such as wavelet-based scalograms.

### 4.10. Future Work and Clinical Implications

Future work will compare STFT with wavelet-based scalograms and other multi-resolution representations. We also plan to explore imbalance-aware learning strategies to improve N1 and N3 classification and to evaluate the framework on additional independent wearable datasets. From a clinical perspective, wearable sleep staging may be useful for long-term sleep monitoring and screening. However, the lower performance for fine-grained stages such as N1 and N3 suggests that these systems should complement, rather than replace, PSG-based clinical assessment.

## 5. Conclusions

In this study, we investigated how signal representation, wearable modality selection, multimodal fusion, acquisition setting, and training cohort size influence wearable sleep-stage classification across three-, four-, and five-class settings. Among the evaluated modalities, cardiovascular signals provided the strongest stage-discriminative information, particularly for REM, while accelerometry contributed complementary movement information, especially for Wake detection. STFT-based representations generally provided stronger class-balanced performance than handcrafted features, whereas combining the two representations yielded only modest additional gains. Importantly, increasing the number of modalities or introducing more complex fusion strategies did not consistently improve Macro-F1, indicating that additional model and sensor complexity is beneficial only when it contributes complementary stage-specific information. The MESA experiments further demonstrated that acquisition conditions and training cohort size can substantially influence performance. Increasing the number and diversity of training subjects improved classification across all sleep-stage granularities; however, N1 and N3 remained comparatively difficult across both DREAMT and MESA. These findings indicate that fine-grained NREM discrimination remains a persistent challenge for sleep staging based on peripheral wearable signals. Overall, the results suggest that effective wearable sleep staging depends more on selecting informative physiological signals and appropriate representations than on simply increasing the number of sensors or model complexity.

## Figures and Tables

**Table 1:** Demographic and sleep characteristics of the datasets. Values are reported as mean ± standard deviation unless otherwise specified.

Variable	DREAMT	MESA-100	MESA-All
Participants included, *n*	99	100	2002
Age, years	56.43±16.53	69.42±8.96	69.36±9.11
Female participants, *n* (%)	54 (54.5%)	50 (50.0%)	1080 (53.9%)
Male participants, *n* (%)	45 (45.5%)	50 (50.0%)	922 (46.1%)
Body mass index, kg/m^2^	33.41±8.26	28.49±5.45	28.64±5.54
Total sleep time, min	299.65±76.74	358.06±87.50	362.87±80.10
Sleep onset latency, min	23.54±22.09	53.38±48.21	45.45±41.37
Wake after sleep onset, min	71.76±55.60	104.57±67.44	94.29±64.70
Sleep efficiency, %	74.41±17.89	74.32±14.98	75.96±13.17
Apnea–hypopnea index, events/h	20.52±23.82	21.28±19.56	23.98±19.38

**Table 2:** Sleep-stage label mappings for the three-, four-, and five-class classification tasks.

Original Label	Five-Class	Four-Class	Three-Class
Wake	Wake	Wake	Wake
N1	N1	Light sleep	NREM
N2	N2	Light sleep	NREM
N3	N3	Deep sleep	NREM
REM	REM	REM	REM

**Table 3:** Distribution of valid 30 s epochs after label mapping for the five-, four-, and three-class sleep-staging tasks.

Classification Task	Sleep-Stage Class	Number of Epochs
Five-class	Wake	19,971
	N1	8819
	N2	39,686
	N3	2661
	REM	8331
	Total	79,468
Four-class	Wake	19,971
	Light sleep (N1 + N2)	48,505
	Deep sleep (N3)	2661
	REM	8331
	Total	79,468
Three-class	Wake	19,971
	NREM (N1 + N2 + N3)	51,166
	REM	8331
	Total	79,468

**Table 4:** Summary of the handcrafted feature representation.

Modality	Feature Groups	Count
Cardiac	BVP morphology, IBI, HR, and PRV	37
ACC	Movement, orientation, temporal, and frequency-domain features	41
EDA	Statistical, tonic, phasic, and response features	20
Temperature	Statistical, trend, and temporal-context features	14
Total		112

**Table 5:** Training configuration of the individual branches and the proposed fusion model.

Configuration	STFT U-Net	Handcrafted CNN–BiGRU	Fusion Head
Optimizer	Adam	AdamW	AdamW
Initial learning rate	$10^{-3}$	$10^{-3}$	$10^{-3}$
Weight decay	–	$10^{-4}$	$10^{-4}$
Maximum epochs	100	30	40
Batch size	2	256	1024
Loss function	Effective-number-weighted cross-entropy	Effective-number-weighted cross-entropy	Effective-number-weighted cross-entropy
Weighting parameter β	0.999	0.999	0.999
Selection metric	Validation Macro-F1	Validation Macro-F1	Validation Macro-F1

**Table 6:** Comparison of accelerometry representations for four-class sleep-stage classification.

ACC Representation	Accuracy	Kappa	Macro-F1
Average of axis log-spectra	0.5587±0.0675	0.2816±0.0581	0.4185±0.0163
Magnitude	**0.5969 ± 0.0301**	0.2997±0.0494	0.4248±0.0416
Separate XYZ channels	0.5572±0.0807	0.2875±0.0880	0.4096±0.0540

Bold values indicate the best performance for each evaluation metric.

**Table 7:** Ablation study for the four-class handcrafted feature representation.

Model	Accuracy	Cohen’s κ	Macro-F1
Random Forest	0.6632	0.2399	0.3154
RF–HMM	**0.6647**	0.2203	0.3052
BiGRU	0.6489	0.3265	0.4115
CNN–BiGRU	0.6612	**0.3475**	**0.4251**
TCN	0.6476	0.3290	0.4180
BiGRU–CRF	0.6602	0.3398	0.4179

Bold values indicate the best performance for each evaluation metric.

**Table 8:** Performance comparison of handcrafted, STFT-based, and hybrid feature representations across the 3-, 4-, and 5-class sleep-stage classification tasks.

Task	Feature Representation	Accuracy	Cohen’s κ	Macro-F1
3-Class	Handcrafted BVP + ACC	0.6816 ± 0.0198	0.3271 ± 0.0433	0.5057 ± 0.0249
	STFT BVP + ACC	0.7365 ± 0.0142	0.4788 ± 0.0368	0.6404 ± 0.0344
	Hybrid fusion	**0.7517 ± 0.0103**	**0.4970 ± 0.0316**	**0.6476 ± 0.0343**
4-Class	Handcrafted BVP + ACC	0.6547 ± 0.0131	0.3194 ± 0.0410	0.4005 ± 0.0353
	STFT BVP + ACC	0.6507 ± 0.0278	0.3956 ± 0.0354	0.5083 ± 0.0320
	Hybrid fusion	**0.6606 ± 0.0229**	**0.4035 ± 0.0361**	**0.5108 ± 0.0338**
5-Class	Handcrafted BVP + ACC	0.5683 ± 0.0327	0.3099 ± 0.0506	0.3366 ± 0.0281
	STFT BVP + ACC	0.5459 ± 0.0398	0.3561 ± 0.0379	0.4376 ± 0.0244
	Hybrid fusion	**0.5835 ± 0.0282**	**0.3833 ± 0.0339**	**0.4464 ± 0.0235**

Bold values indicate the best performance for each evaluation metric.

**Table 9:** Performance comparison of single and fusion modalities for three-class sleep-stage classification using handcrafted features.

Handcrafted Feature Set	Accuracy	Cohen’s κ	Macro-F1	Wake F1	NREM F1	REM F1
ACC	0.6818±0.0290	0.3412±0.0715	0.4924±0.0347	0.6260±0.0517	0.7689±0.0322	0.0823±0.0796
BVP	0.6807±0.0164	0.3140±0.0267	0.4721±0.0244	0.5661±0.0464	0.7784±0.0302	0.0720±0.0656
EDA	0.6621±0.0412	0.2504±0.0479	0.4267±0.0196	0.4943±0.0502	0.7697±0.0361	0.0161±0.0267
**HR**	0.7016±0.0112	0.4106±0.0337	0.6011±0.0303	0.5851±0.0383	0.7866±0.0167	0.4315±0.0792
PRV	0.6708±0.0310	0.3418±0.0569	0.5287±0.0265	0.5648±0.0645	0.7633±0.0385	0.2580±0.0832
TEMP	0.6412±0.0235	0.2115±0.0647	0.4166±0.0327	0.4498±0.0969	0.7519±0.0308	0.0481±0.0403
BVP + ACC	0.6816±0.0198	0.3271±0.0433	0.5057±0.0249	0.6000±0.0579	0.7748±0.0308	0.1423±0.0509
BVP + HR	0.7012±0.0241	0.3958±0.0768	0.5833±0.0649	0.5712±0.0860	0.7886±0.0117	0.3901±0.1408
BVP + PRV	0.6770±0.0205	0.3406±0.0136	0.5113±0.0336	0.5705±0.0272	0.7729±0.0263	0.1904±0.1032
PRV + HR	0.6985±0.0204	0.3779±0.0439	0.5744±0.0248	0.5585±0.0545	0.7884±0.0223	0.3763±0.0542
ACC + EDA + TEMP	0.6879±0.0157	0.3345±0.0461	0.4946±0.0336	0.6007±0.0542	0.7780±0.0212	0.1050±0.0833
BVP + PRV + HR	0.6944±0.0234	0.3733±0.0571	0.5699±0.0303	0.5623±0.0680	0.7848±0.0280	0.3626±0.0418
BVP + PRV + HR + ACC	0.7035±0.0122	0.3872±0.0357	0.5485±0.0300	0.6100±0.0452	0.7930±0.0077	0.2425±0.0881
BVP + PRV + HR + EDA	0.6777±0.0258	0.3440±0.0373	0.5462±0.0237	0.5359±0.0654	0.7736±0.0342	0.3292±0.0356
BVP + PRV + HR + TEMP	0.6940±0.0268	0.3674±0.0301	0.5542±0.0221	0.5546±0.0352	0.7893±0.0323	0.3187±0.0451
BVP + PRV + HR + ACC + EDA	0.7146±0.0057	0.4074±0.0371	0.5699±0.0197	0.6217±0.0423	0.7981±0.0103	0.2899±0.0636
BVP + PRV + HR + ACC + TEMP	0.7093±0.0139	0.4069±0.0412	0.5772±0.0213	0.6169±0.0491	0.7948±0.0105	0.3200±0.0456
BVP + PRV + HR + EDA + TEMP	0.6762±0.0146	0.3631±0.0186	0.5539±0.0087	0.5689±0.0366	0.7695±0.0226	0.3234±0.0413
BVP + PRV + HR + ACC + EDA + TEMP	0.6987±0.0121	0.3760±0.0349	0.5416±0.0320	0.6026±0.0408	0.7862±0.0092	0.2358±0.1100

Bold values indicate the best performance for each evaluation metric.

**Table 10:** Performance comparison of single and fusion modalities for four-class sleep-stage classification using handcrafted features.

Feature Combination	Accuracy	Cohen’s κ	Macro-F1	Wake F1	Light F1	Deep F1	REM F1
ACC	0.6638±0.0196	0.3148±0.0522	0.3800±0.0464	0.6067±0.0655	0.7595±0.0251	0.0909±0.0835	0.0629±0.0629
BVP	0.6226±0.0291	0.2595±0.0442	0.3537±0.0219	0.5573±0.0466	0.7304±0.0302	0.0432±0.0598	0.0839±0.0598
EDA	0.6456±0.0304	0.2567±0.0329	0.3310±0.0230	0.5325±0.0441	0.7518±0.0329	0.0010±0.0023	0.0387±0.0722
**HR**	0.6520±0.0370	0.3538±0.0617	0.4510±0.0218	0.5732±0.0510	0.7398±0.0353	0.0735±0.0665	0.4174±0.0878
PRV	0.6424±0.0397	0.2905±0.0460	0.3780±0.0242	0.5443±0.0368	0.7423±0.0391	0.0022±0.0049	0.2231±0.0678
TEMP	0.6179±0.0244	0.1868±0.0538	0.3041±0.0250	0.4382±0.0904	0.7326±0.0341	0.0000±0.0000	0.0455±0.0652
BVP + HR	0.6520±0.0299	0.3445±0.0626	0.4393±0.0425	0.5710±0.0693	0.7443±0.0254	0.1042±0.0931	0.3377±0.1085
BVP + PRV	0.6322±0.0194	0.2995±0.0493	0.4068±0.0488	0.5676±0.0424	0.7297±0.0240	0.1203±0.1122	0.2095±0.0938
PRV + HR	0.6320±0.0187	0.3175±0.0429	0.4088±0.0260	0.5700±0.0339	0.7207±0.0552	0.0478±0.0835	0.2966±0.1188
ACC + EDA + TEMP	0.6489±0.0183	0.3229±0.0385	0.3913±0.0301	0.6294±0.0365	0.7350±0.0317	0.0214±0.0332	0.1795±0.0733
BVP + PRV + HR	0.6508±0.0272	0.3364±0.0610	0.4207±0.0458	0.5796±0.0635	0.7402±0.0393	0.0425±0.0522	0.3204±0.1230
BVP + PRV + HR + ACC	0.6687±0.0320	0.3598±0.0544	0.4429±0.0318	0.6146±0.0546	0.7554±0.0330	0.1023±0.0782	0.2993±0.0832
BVP + PRV + HR + EDA	0.6521±0.0203	0.3337±0.0582	0.4296±0.0376	0.5634±0.0610	0.7495±0.0117	0.0826±0.0754	0.3229±0.0794
BVP + PRV + HR + TEMP	0.6519±0.0144	0.3192±0.0266	0.3973±0.0409	0.5599±0.0504	0.7501±0.0221	0.0218±0.0473	0.2572±0.1508
BVP + PRV + HR + ACC + EDA	0.6585±0.0278	0.3435±0.0404	0.4164±0.0413	0.6092±0.0477	0.7511±0.0180	0.0729±0.1244	0.2322±0.1339
BVP + PRV + HR + ACC + TEMP	0.6716±0.0268	0.3526±0.0657	0.4249±0.0520	0.6096±0.0632	0.7619±0.0166	0.0455±0.0770	0.2825±0.1160
BVP + PRV + HR + EDA + TEMP	0.6322±0.0176	0.3230±0.0227	0.4207±0.0226	0.5671±0.0163	0.7255±0.0240	0.0427±0.0731	0.3476±0.0528
BVP + PRV + HR + ACC + EDA + TEMP	0.6612±0.0242	0.3475±0.0579	0.4251±0.0352	0.6086±0.0523	0.7528±0.0111	0.0400±0.0793	0.2991±0.0399

Bold values indicate the best performance for each evaluation metric.

**Table 11:** Performance comparison of single and fusion modalities for five-class sleep-stage classi- fication using handcrafted features.

Handcrafted Feature Set	Accuracy	Cohen’s κ	Macro-F1	Wake F1	N1 F1	N2 F1	N3 F1	REM F1
ACC	0.5644±0.0639	0.2788±0.0697	0.3054±0.0478	0.6128±0.0574	0.0672±0.0455	0.6745±0.0557	0.0710±0.1348	0.1018±0.0887
BVP	0.5302±0.0404	0.2519±0.0562	0.3008±0.0193	0.5840±0.0710	0.0725±0.0341	0.6512±0.0333	0.0908±0.1318	0.1056±0.0714
EDA	0.5488±0.0256	0.2109±0.0474	0.2460±0.0219	0.5164±0.0852	0.0003±0.0007	0.6739±0.0299	0.0006±0.0014	0.0391±0.0656
HR	0.5772±0.0270	0.3267±0.0431	0.3514±0.0294	0.5857±0.0498	0.0483±0.0442	0.6895±0.0225	0.0222±0.0272	0.4114±0.0918
PRV	0.5422±0.0175	0.2647±0.0388	0.3147±0.0180	0.5435±0.0576	0.0570±0.0394	0.6609±0.0079	0.0021±0.0031	0.3099±0.0719
TEMP	0.5242±0.0223	0.1807±0.0585	0.2535±0.0317	0.4645±0.0300	0.0000±0.0000	0.6531±0.0180	0.0240±0.0582	0.1238±0.0854
BVP + ACC	0.5683±0.0327	0.3099±0.0506	0.3366±0.0281	0.6511±0.0518	0.0970±0.0317	0.6725±0.0272	0.0542±0.0804	0.2084±0.0743
BVP + HR	0.5826±0.0517	0.3296±0.0759	0.3542±0.0339	0.5863±0.0916	0.0453±0.0231	0.6933±0.0428	0.0820±0.1116	0.3642±0.1095
BVP + PRV	0.5481±0.0447	0.2831±0.0637	0.3273±0.0222	0.5941±0.0652	0.0667±0.0426	0.6692±0.0363	0.0712±0.0661	0.2450±0.0733
PRV + HR	0.5720±0.0168	0.3074±0.0269	0.3593±0.0285	0.5808±0.0235	0.0645±0.0299	0.6854±0.0181	0.1294±0.0909	0.3406±0.0669
ACC + EDA + TEMP	0.5851±0.0286	0.3085±0.0264	0.3146±0.0166	0.6408±0.0298	0.0602±0.0446	0.6963±0.0263	0.0083±0.0081	0.1673±0.0849
BVP + PRV + HR	0.5914±0.0301	0.3293±0.0533	0.3508±0.0233	0.6008±0.0650	0.0717±0.0425	0.7028±0.0194	0.0263±0.0380	0.3522±0.0770
BVP + PRV + HR + ACC	0.5861±0.0269	0.3246±0.0451	0.3575±0.0435	0.6269±0.0460	0.0601±0.0674	0.6940±0.0185	0.1081±0.1135	0.2986±0.0791
BVP + PRV + HR + EDA	0.5552±0.0379	0.2921±0.0527	0.3385±0.0200	0.5718±0.0524	0.0572±0.0562	0.6687±0.0414	0.0791±0.0896	0.3159±0.0550
BVP + PRV + HR + TEMP	0.5658±0.0366	0.2918±0.0468	0.3143±0.0415	0.5598±0.0414	0.0585±0.0593	0.6698±0.0322	0.0000±0.0000	0.2863±0.1511
**BVP + PRV + HR + ACC + EDA**	0.5858±0.0198	0.3392±0.0271	0.3679±0.0254	0.6268±0.0555	0.0790±0.0592	0.6943±0.0101	0.1044±0.0866	0.3348±0.0444
BVP + PRV + HR + ACC + TEMP	0.5793±0.0322	0.3223±0.0395	0.3559±0.0247	0.6184±0.0288	0.0894±0.0440	0.6940±0.0228	0.0340±0.0420	0.3436±0.0794
BVP + PRV + HR + EDA + TEMP	0.5436±0.0263	0.2808±0.0571	0.3302±0.0272	0.5466±0.0445	0.0645±0.0276	0.6640±0.0233	0.0411±0.0748	0.3346±0.0474
BVP + PRV + HR + ACC + EDA + TEMP	0.5749±0.0421	0.3314±0.0551	0.3673±0.0335	0.6286±0.0355	0.0832±0.0365	0.6834±0.0337	0.0747±0.0748	0.3666±0.0706

Bold values indicate the best performance for each evaluation metric.

**Table 12:** Performance comparison of modalities for STFT-based three-class sleep-stage classification.

STFT Input Modalities	Accuracy	Cohen’s κ	Macro-F1	Wake F1	NREM F1	REM F1
BVP only	0.7274±0.0079	0.4647±0.0386	0.6255±0.0292	0.6223±0.0460	0.8099±0.0150	0.4442±0.0753
ACC magnitude only	0.6696±0.0321	0.3348±0.0601	0.4817±0.0396	0.6095±0.0647	0.7597±0.0310	0.0758±0.0735
**BVP + ACC magnitude**	0.7365±0.0142	0.4788±0.0368	0.6404±0.0344	0.6473±0.0303	0.8109±0.0154	0.4631±0.0875

Bold values indicate the best performance for each evaluation metric.

**Table 13:** Performance comparison of modalities for STFT-based four-class sleep-stage classification.

STFT Input Modalities	Accuracy	Cohen’s κ	Macro-F1	Wake F1	Light F1	Deep F1	REM F1
BVP only	0.6386±0.0374	0.3904±0.0459	0.4996±0.0192	0.6341±0.0494	0.7060±0.0473	0.2037±0.0592	0.4543±0.1173
ACC magnitude only	0.5969±0.0301	0.2997±0.0494	0.4248±0.0416	0.6027±0.0490	0.6856±0.0262	0.1612±0.1328	0.2496±0.0253
**BVP + ACC magnitude**	0.6507±0.0278	0.3956±0.0354	0.5083±0.0320	0.6517±0.0356	0.7165±0.0447	0.2156±0.1113	0.4493±0.0630

Bold values indicate the best performance for each evaluation metric.

**Table 14:** Performance comparison of modalities for STFT-based five-class sleep-stage classification.

STFT Input Modalities	Accuracy	Cohen’s κ	Macro-F1	Wake F1	N1 F1	N2 F1	N3 F1	REM F1
**BVP only**	0.5513±0.0237	0.3607±0.0250	0.4520±0.0184	0.6164±0.0624	0.2531±0.0409	0.6567±0.0522	0.2328±0.0514	0.5009±0.0901
ACC magnitude only	0.4990±0.0356	0.2796±0.0364	0.3731±0.0236	0.6031±0.0422	0.2188±0.0222	0.6078±0.0606	0.1869±0.0691	0.2492±0.0520
BVP + ACC magnitude	0.5459±0.0398	0.3561±0.0379	0.4376±0.0244	0.6240±0.0422	0.2578±0.0139	0.6525±0.0508	0.2186±0.0598	0.4350±0.0981

Bold values indicate the best performance for each evaluation metric.

**Table 15:** Fusion performance for three-class sleep-stage classification using BVP–ACC STFT and handcrafted modality models.

Fusion Configuration	Accuracy	Cohen’s κ	Macro-F1	Wake F1	NREM F1	REM F1
None (BVP–ACC STFT only)	0.7365±0.0142	0.4788±0.0368	0.6404±0.0344	0.6473±0.0303	0.8109±0.0154	0.4631±0.0875
BVP	0.7419±0.0185	0.4813±0.0396	0.6334±0.0409	0.6562±0.0285	0.8153±0.0224	0.4286±0.1064
ACC	0.7435±0.0131	0.4899±0.0374	0.6441±0.0359	0.6593±0.0357	0.8159±0.0134	0.4570±0.0912
PRV	0.7462±0.0156	0.4877±0.0317	0.6414±0.0336	0.6530±0.0302	0.8196±0.0191	0.4516±0.1005
HR	0.7439±0.0241	0.4798±0.0543	0.6401±0.0484	0.6458±0.0304	0.8180±0.0234	0.4566±0.1095
EDA	0.7431±0.0159	0.4839±0.0372	0.6411±0.0353	0.6492±0.0316	0.8174±0.0156	0.4567±0.0924
Temperature	0.7407±0.0170	0.4809±0.0375	0.6401±0.0353	0.6487±0.0302	0.8148±0.0183	0.4568±0.0903
**BVP + ACC**	0.7517±0.0103	0.4970±0.0316	0.6476±0.0343	0.6647±0.0284	0.8229±0.0159	0.4553±0.0948
BVP + PRV + HR	0.7405±0.0116	0.4717±0.0083	0.6308±0.0173	0.6418±0.0264	0.8166±0.0197	0.4339±0.0639
BVP + PRV + HR + ACC	0.7485±0.0128	0.4939±0.0352	0.6434±0.0355	0.6622±0.0297	0.8217±0.0135	0.4463±0.0908
BVP + PRV + HR + EDA	0.7432±0.0093	0.4844±0.0313	0.6451±0.0304	0.6473±0.0323	0.8165±0.0144	0.4716±0.0822
BVP + PRV + HR + Temperature	0.7347±0.0282	0.4604±0.0405	0.6196±0.0286	0.6346±0.0330	0.8137±0.0277	0.4106±0.0570
BVP + PRV + HR + ACC + EDA	0.7458±0.0094	0.4867±0.0377	0.6395±0.0351	0.6582±0.0363	0.8189±0.0107	0.4413±0.0879
BVP + PRV + HR + ACC + Temperature	0.7473±0.0064	0.4830±0.0166	0.6318±0.0192	0.6602±0.0270	0.8218±0.0123	0.4135±0.0607
BVP + PRV + HR + EDA + Temperature	0.7433±0.0124	0.4841±0.0260	0.6420±0.0303	0.6496±0.0202	0.8172±0.0167	0.4591±0.0856
BVP + PRV + HR + ACC + EDA + Temperature	0.7469±0.0135	0.4778±0.0111	0.6265±0.0294	0.6556±0.0225	0.8207±0.0168	0.4032±0.0912

Bold values indicate the best performance for each evaluation metric.

**Table 16:** Fusion performance for four-class sleep-stage classification using BVP–ACC STFT and handcrafted modality models.

Fusion Configuration	Accuracy	Cohen’s κ	Macro-F1	Wake F1	Light F1	Deep F1	REM F1
None (BVP–ACC STFT only)	0.6507±0.0278	0.3956±0.0354	0.5083±0.0320	0.6517±0.0356	0.7165±0.0447	0.2156±0.1113	0.4493±0.0630
BVP	0.6628±0.0269	0.4049±0.0359	0.5096±0.0333	0.6545±0.0390	0.7314±0.0365	0.2019±0.0929	0.4506±0.0616
ACC	0.6654±0.0302	0.4092±0.0426	0.5136±0.0349	0.6556±0.0419	0.7328±0.0391	0.2115±0.1108	0.4546±0.0630
PRV	0.6660±0.0226	0.4071±0.0401	0.5072±0.0312	0.6501±0.0320	0.7363±0.0244	0.1878±0.0950	0.4546±0.0699
HR	0.6862±0.0229	0.4097±0.0531	0.4868±0.0418	0.6394±0.0435	0.7647±0.0214	0.1292±0.0272	0.4140±0.1232
EDA	0.6641±0.0270	0.4051±0.0381	0.5088±0.0328	0.6528±0.0372	0.7332±0.0337	0.1961±0.1007	0.4532±0.0622
Temperature	0.6672±0.0307	0.4071±0.0410	0.5108±0.0344	0.6519±0.0387	0.7356±0.0422	0.2031±0.1127	0.4527±0.0632
BVP + ACC	0.6606±0.0257	0.4035±0.0361	0.5108±0.0338	0.6539±0.0388	0.7287±0.0356	0.2089±0.1033	0.4516±0.0611
BVP + PRV + HR	0.6687±0.0229	0.4120±0.0341	0.5144±0.0337	0.6559±0.0348	0.7365±0.0346	0.2086±0.1093	0.4564±0.0656
BVP + PRV + HR + ACC	0.6704±0.0246	0.4143±0.0413	0.5132±0.0344	0.6579±0.0400	0.7387±0.0321	0.1960±0.1163	0.4603±0.0666
BVP + PRV + HR + EDA	0.6705±0.0228	0.4124±0.0375	0.5112±0.0295	0.6521±0.0341	0.7415±0.0254	0.1921±0.0919	0.4589±0.0538
**BVP + PRV + HR + Temperature**	0.6709±0.0344	0.4135±0.0294	0.5178±0.0379	0.6518±0.0428	0.7376±0.0500	0.2201±0.1248	0.4615±0.0631
BVP + PRV + HR + ACC + EDA	0.6664±0.0230	0.4067±0.0403	0.5059±0.0311	0.6542±0.0380	0.7364±0.0287	0.1781±0.1052	0.4548±0.0689
BVP + PRV + HR + ACC + Temperature	0.6749±0.0210	0.4172±0.0391	0.5134±0.0336	0.6572±0.0393	0.7440±0.0259	0.1898±0.0950	0.4625±0.0650
BVP + PRV + HR + EDA + Temperature	0.6666±0.0269	0.4095±0.0401	0.5098±0.0331	0.6546±0.0344	0.7368±0.0295	0.1922±0.0933	0.4556±0.0644
BVP + PRV + HR + ACC + EDA + Temperature	0.6782±0.0275	0.4206±0.0461	0.5103±0.0310	0.6571±0.0368	0.7477±0.0289	0.1675±0.1026	0.4691±0.0705

Bold values indicate the best performance for each evaluation metric.

**Table 17:** Fusion performance for five-class sleep-stage classification using BVP–ACC STFT and handcrafted modality models.

Fusion Configuration	Accuracy	Cohen’s κ	Macro-F1	Wake F1	N1 F1	N2 F1	N3 F1	REM F1
None (BVP–ACC STFT only)	0.5459±0.0398	0.3561±0.0379	0.4376±0.0244	0.6240±0.0422	0.2578±0.0139	0.6525±0.0508	0.2186±0.0598	0.4350±0.0981
BVP	0.5868±0.0136	0.3800±0.0259	0.4411±0.0212	0.6493±0.0341	0.2285±0.0285	0.6966±0.0202	0.1989±0.0574	0.4321±0.0945
ACC	0.5814±0.0340	0.3754±0.0386	0.4384±0.0249	0.6426±0.0402	0.2329±0.0272	0.6877±0.0401	0.1973±0.0523	0.4316±0.0980
PRV	0.5676±0.0195	0.3686±0.0306	0.4366±0.0260	0.6286±0.0360	0.2434±0.0274	0.6804±0.0186	0.1887±0.0780	0.4422±0.1045
HR	0.5858±0.0190	0.3864±0.0352	0.4415±0.0287	0.6408±0.0476	0.2274±0.0481	0.6980±0.0100	0.1829±0.0860	0.4585±0.1187
EDA	0.5722±0.0353	0.3656±0.0370	0.4337±0.0242	0.6366±0.0392	0.2288±0.0315	0.6818±0.0347	0.1919±0.0708	0.4293±0.0916
Temperature	0.5765±0.0222	0.3691±0.0354	0.4366±0.0252	0.6345±0.0415	0.2222±0.0436	0.6875±0.0245	0.2035±0.0572	0.4354±0.0939
**BVP + ACC**	0.5835±0.0282	0.3833±0.0339	0.4464±0.0235	0.6527±0.0366	0.2434±0.0196	0.6892±0.0349	0.2077±0.0441	0.4389±0.1003
BVP + PRV + HR	0.5785±0.0288	0.3771±0.0315	0.4424±0.0194	0.6371±0.0387	0.2340±0.0312	0.6884±0.0307	0.2005±0.0624	0.4521±0.1058
BVP + PRV + HR + ACC	0.5850±0.0202	0.3806±0.0281	0.4368±0.0187	0.6516±0.0320	0.2242±0.0392	0.6929±0.0219	0.1717±0.0408	0.4438±0.1035
BVP + PRV + HR + EDA	0.5741±0.0283	0.3747±0.0340	0.4401±0.0207	0.6348±0.0410	0.2391±0.0258	0.6832±0.0259	0.1945±0.0669	0.4491±0.1058
BVP + PRV + HR + Temperature	0.5824±0.0245	0.3789±0.0294	0.4399±0.0204	0.6391±0.0339	0.2238±0.0303	0.6937±0.0257	0.1939±0.0480	0.4490±0.1083
BVP + PRV + HR + ACC + EDA	0.5913±0.0319	0.3857±0.0325	0.4304±0.0226	0.6502±0.0441	0.2124±0.0484	0.6983±0.0295	0.1506±0.0817	0.4405±0.0969
BVP + PRV + HR + ACC + Temperature	0.5898±0.0316	0.3860±0.0377	0.4304±0.0264	0.6533±0.0393	0.2219±0.0335	0.6972±0.0327	0.1296±0.0702	0.4502±0.1139
BVP + PRV + HR + EDA + Temperature	0.5787±0.0279	0.3769±0.0305	0.4399±0.0214	0.6413±0.0331	0.2239±0.0358	0.6860±0.0346	0.1943±0.0251	0.4539±0.1020
BVP + PRV + HR + ACC + EDA + Temperature	0.5885±0.0366	0.3870±0.0359	0.4399±0.0193	0.6552±0.0345	0.2288±0.0321	0.6925±0.0396	0.1648±0.0273	0.4582±0.0951

Bold values indicate the best performance for each evaluation metric.

**Table 18:** Performance comparison of fusion strategies.

Fusion Strategy	Accuracy	Cohen’s κ	Macro-F1
Frozen concatenation	0.6687±0.0229	0.4120±0.0341	**0.5144 ± 0.0337**
Joint fine-tuning	0.6949±0.0333	0.4174±0.0497	0.4908±0.0388
Gated residual fusion	0.6940±0.0339	0.4176±0.0428	0.4993±0.0379

Bold values indicate the best performance for each evaluation metric.

**Table 19:** Performance comparison on the MESA-100 and full MESA cohorts for 3-, 4-, and 5-class sleep-stage classification.

Task	Cohort	Accuracy	Cohen’s κ	Macro-F1
3-Class	MESA-100	0.7613	0.5907	0.7059
	Full MESA	**0.8600**	**0.7705**	**0.8430**
4-Class	MESA-100	0.7288	0.5759	0.6564
	Full MESA	**0.7802**	**0.6600**	**0.7101**
5-Class	MESA-100	0.6258	0.4925	0.5515
	Full MESA	**0.7082**	**0.5942**	**0.6265**

Bold values indicate the best performance for each evaluation metric.

**Table 20:** Performance comparison on the MESA-100 and full MESA cohorts for five classes in terms of per-stage F1 scores.

Cohort	Wake	N1	N2	N3	REM
MESA-100	0.8022	0.3090	0.5822	0.4421	0.6220
Full MESA	**0.8622**	**0.3624**	**0.6811**	**0.4823**	**0.7443**

Bold values indicate the best performance for each evaluation metric.

## Data Availability

The datasets analyzed in this study are available from existing data repositories. The DREAMT dataset is publicly available through PhysioNet. The MESA Sleep dataset is available through the National Sleep Research Resource (NSRR), subject to the applicable data-access requirements and approval. No new participant data were collected as part of this study.

## Abbreviations

The following abbreviations are used in this manuscript:

ACC	Accelerometry
AHI	Apnea–Hypopnea Index
AASM	American Academy of Sleep Medicine
BVP	Blood Volume Pulse
CNN	Convolutional Neural Network
CRF	Conditional Random Field
EDA	Electrodermal Activity
HR	Heart Rate
HMM	Hidden Markov Model
IBI	Interbeat Interval
MESA	Multi-Ethnic Study of Atherosclerosis
NREM	Non-Rapid Eye Movement
NSRR	National Sleep Research Resource
PPG	Photoplethysmography
PRV	Pulse Rate Variability
PSG	Polysomnography
REM	Rapid Eye Movement
RF	Random Forest
STFT	Short-Time Fourier Transform
TCN	Temporal Convolutional Network
